# “And then the person sort of just drops off the radar…”: barriers in the transition from hospital to community-based care among survivors of intimate partner violence in Metropolitan Atlanta

**DOI:** 10.3389/fpubh.2024.1332779

**Published:** 2024-05-22

**Authors:** Dabney P. Evans, Jocelyn Pawcio, Kathryn Wyckoff, Lee Wilkers

**Affiliations:** Hubert Department of Global Health, Emory University, Atlanta, GA, United States

**Keywords:** intimate partner, community based organization, coordinated care, violence, hospital

## Abstract

**Introduction:**

Hospitals and community-based organizations (CBOs) provide the service-base for survivors of intimate partner violence (IPV), particularly those in acute crisis. Both settings face discrete challenges in meeting survivors’ needs. In hospitals these challenges include the pressures of a fast-paced work setting, and a lack of trauma-informed and survivor-centered care. Connections to community care are often unmeasured, with relatively little known about best practices. Often IPV survivors who receive hospital care fail to connect with community-based services after discharge. Despite the critical role of CBOs in supporting IPV survivors, there is limited research examining the perspectives and insights of CBO staff on the challenges and opportunities for improving care coordination with hospitals. The purpose of this study was to address this knowledge gap by characterizing CBO staff perceptions of IPV care coordination between hospital and community-based organizations in Metropolitan Atlanta.

**Methods:**

We used a qualitative study design to conduct a cross-sectional examination of the perceptions and experiences of staff working at CBOs serving IPV survivors in Metropolitan Atlanta, Georgia. The adapted in-depth interview (IDI) guide was used to explore: (1) IPV survivor experiences; (2) Survivors’ needs when transitioning from hospital to community-based care; (3) Barriers and facilitators to IPV care coordination; and (4) Ideas on how to improve care coordination. Data analysis consisted of a thematic analysis using MAXQDA Analytics Pro 2022.

**Results:**

Participants (*N* = 14) included 13 women and one man who were staff of CBOs serving IPV survivors in Metropolitan Atlanta. CBO staff perceived that: (1) IPV survivors face individual-, organizational-, and systems-level barriers during help seeking and service provision; (2) Care coordination between hospitals and CBOs is limited due to siloed care provision; and (3) Care coordination can be improved through increased bidirectional efforts.

**Conclusion:**

Our findings highlight the multi-level barriers IPV survivors face in accessing community-based care following medical care, the limitations of existing hospital-CBO coordination, and opportunities for improvement from the perspectives of CBO staff. Participants identified silos and inconsistent communication/relationships between hospital and CBOs as major barriers to care connections. They also suggested warm handoffs and a Family Justice Center to support care connection.

## Background

Intimate partner violence (IPV), emotional, physical, and/or sexual violence tactics perpetrated by current or previous intimate partners, impacts up to 753 million women worldwide (1, 2). In the US, IPV is common, with more than 35% of women and 28% of men reporting lifetime IPV (3). Emergency departments (EDs) provide vital and often life-saving care to people experiencing IPV. Because of social stigma, self-blame, and the emotional trauma associated with relationship violence, those experiencing abuse may not seek health care immediately and may do so primarily after serious physical injury (4–9). As a result, EDs may serve as the first point of contact for IPV survivors who make up at least 5% of all ED visits nationwide—with many cases going undetected due to the limits of using diagnostic codes as the exclusive markers of abuse (10–14).

Where EDs meet the acute medical care needs of IPV survivors, networks of community-based organizations (CBOs) specializing in violence response support the short- and long-term needs of people leaving violent relationships by connecting survivors to an array of essential social services such as safe housing, legal assistance, and psychological counseling (14, 15). Yet both settings face discrete challenges in meeting survivors’ needs. In hospitals these challenges include the pressures of a fast-paced work setting, staff shortages, provider misperceptions of IPV experiences, and lack of trauma-informed and survivor-centered care (16). IPV screening, service referral, and connections to community care are often unmeasured, with relatively little known about efficacy or best practices in hospital settings (15, 17–21). Concurrently, CBOs face funding and sustainability challenges, demand for services which routinely outpaces availability, and the need for comprehensive cross-sectoral services to meet the co-occurring social support needs of survivors (22–24). Taken together, hospitals and CBOs provide the service-base for IPV survivors, particularly those in acute crisis where according to one study (*n* = 1,268) nearly 20% of women seeking care across 24 emergency departments had experienced physical violence or severe physical abuse (13).

Often IPV survivors who receive hospital care fail to connect with community-based services after discharge. In a sample (*n* = 245) of hospitalized IPV survivors (97% women; mean age 37 years) discharged from a safety-net hospital in Atlanta in 2019 with sporadic CBO services in the hospital, 40% were discharged with no identified safe shelter (defined as discharge to a shelter, a family/friend, or known safe location without a perpetrator present); only 6% were discharged to placement in a shelter (25). In a related study during the COVID-19 pandemic, after accepting the opportunity to stay in an extended observation unit to optimize the chance for social work intervention, 70.7% of IPV survivors received a safe discharge—including 31% to a shelter—suggesting that increased coordination between hospital- and community-based systems have promise in meeting survivor needs (25). Despite the critical role of CBOs in supporting IPV survivors, there is limited research examining the perspectives and insights of CBO staff on the challenges and opportunities for improving care coordination with hospitals. The purpose of this study was to address this knowledge gap by characterizing CBO staff perceptions of IPV care coordination between hospital and community-based organizations in Metropolitan Atlanta.

## Methods

### Study setting

This study took place in Metropolitan Atlanta, Georgia. Georgia is located in the southern US and is one of only ten states who have opted not to participate Medicaid expansion, a federal program that provides health insurance coverage to poor people (26). In 2022, there were 129,528 crisis calls to Georgia’s certified family violence and sexual assault agencies, a 13% increase from 2021 (27). The GCFV found a 42% increase in family violence-related fatalities in Georgia from 2012 to 2022 (27). These data align with an increase in IPV calls and cases attributed to the COVID-19 pandemic suggesting a “new normal” for family and intimate partner violence in Atlanta (28).

Metropolitan Atlanta consists of eleven counties that are home to eleven million people. Accessing IPV resources can be challenging for survivors given that survivor needs vary and are inherently complex. There are no formally established care coordination programs between Atlanta hospitals and community-based organizations serving IPV survivors. In most hospital settings standard care includes the provision of informational resources with no follow-up. Outside hospital settings, multiple community-based organizations serve survivors of IPV, providing specialized and non-IPV-specific support services. The Georgia Coalition Against Domestic Violence (GCADV) is a statewide coalition that provides a 24-hour hotline and services such as crisis counseling, support groups, and legal assistance, and includes 63 organizations based in Georgia (29). The GCADV coordinates with other organizations within the state and found shelter for over 5,000 survivors and their children in fiscal year 2021 (29); notably in the same year 4,200 survivors and their children were turned away from shelters due to a shortage of beds. The GCADV hotline connects with state-certified shelters, with calls being forwarded to the closest shelter based on area code. The hotline also offers language interpretation for survivors. In addition to shelter, local CBOs offer counseling, legal aid, financial assistance, safety planning, and support groups among other supportive social services (30, 31); some agencies focus on specific populations such as Latinx, South Asian and immigrant survivors (32–34).

### Design

We used a qualitative study design to conduct a cross-sectional examination of the perceptions and experiences of staff working at a community-based organizations (CBOs) serving IPV survivors in Metropolitan Atlanta, Georgia. We were specifically interested in care coordination between hospitals to community-based organizations, and care and interactions between IPV survivors and the professionals serving them. This study focused on the perspectives of individuals working in CBOs and their experiences serving IPV survivors. In-depth interviews (IDIs) were selected for use given the sensitivity of IPV as a topic. Moreover, IDIs support rapport-building and were appropriate for the study given the potential for discussion of experiences serving IPV survivors which might not be disclosed in other settings. The use of IDIs also provided for the protection of confidentiality given power and organizational dynamics within and between CBOs serving IPV survivors. Emory University’s Institutional Review Board deemed this study exempt from review based on its nature as a public health practice.

### Instrument

An existing in-depth interview guide (IDI) was adapted for use among CBO staff. The original guide was used among healthcare professionals providing hospital-based care to IPV survivors in Metropolitan Atlanta during the COVID-19 pandemic (35). Adaptations included a reframing of the guide to CBO settings (e.g., What barriers does your organization face in serving IPV survivors?). The adapted IDI guide consisted of questions to gather perceptions and experiences about several domains: (1) IPV survivor experiences; (2) Survivors’ needs when transitioning from hospital to community-based care; (3) Barriers and facilitators for IPV care coordination; and (4) Ideas on how to improve care coordination between hospital and community-based organizations. The guide was divided into six sections and included 23 questions, including probes. The first section included quantitative demographic information. The second section asked qualitative and quantitative questions about social service employment history. The next section consisted of health and support-seeking behaviors with quantitative and qualitative questions about IPV and what training CBO had staff received. We also asked for an estimate of how many IPV survivors the CBO staff saw within 1 day. Section four revolved around community-based care, the support CBOs offer their clients within 48 hours of intake, and any barriers in serving survivors. The next section consisted of questions about care transitions and the main barriers to care coordination between hospitals and CBOs; we asked participants to estimate the proportion of IPV survivors that they serve who come directly from a hospital to their CBO. We also asked for their insights into any differences between IPV survivors that receive care at a CBO following hospital discharge versus those who do not. In the closing section, participants were asked for suggestions to better respond to IPV and if there were any additional topics they would like to discuss. The second author pilot-tested the adapted IDI guide with members of the research team and public health professionals unaffiliated with the study to gather feedback from practice interviews (*n* = 8). Critiques and edits were incorporated into the final guide, including probing techniques to extract additional information from participants and clarifying questions to avoid confusion.

### Participants and recruitment

To be eligible for study participation, participants must have worked at a CBO serving IPV survivors for at least 6 months. All recruitment took place over email using an electronic flier containing participant eligibility requirements, study information, and contact information for the study team. Initial recruitment occurred in March 2022, following a quarterly meeting of the Georgia Coalition Against Domestic Violence (GCADV), where the first author presented findings from an earlier study. At this time the first author also described the current study and shared the recruitment flier. The study team followed up by emailing the recruitment flier to those attending the meeting with an invitation to participate in the study. Next, using a publicly available list of agencies serving IPV survivors in Metropolitan Atlanta, the study team sent recruitment emails to the Executive Directors of each agency, asking that they share the study recruitment flyer with their staff. Finally, using snowball sampling methods, we asked each participant to recommend up to three individuals they believed could contribute to the study via email referrals. Those who did not respond were contacted via email a total of four times before study exclusion. Individuals who expressed interest via email were asked to schedule an interview for a day and time that worked for them. Next, they were sent a consent form to review before the interview; the consent form explained the study’s purpose in keeping with best practices. They were also sent a Zoom link for the interview and a calendar invite. Verbal consent was obtained before each interview began.

### Data collection and management

Data collection occurred from June through December 2022. Following pilot testing and training, two study team members conducted 14 in-depth interviews. After the second author was sufficiently trained, they continued interviewing independently; the third author was also present to take field notes for some interviews (*n* = 4). To ensure that privacy and safety were maintained, the consent form was reviewed prior to the start of each interview. Interviews were conducted and recorded remotely with permission via Zoom, lasting between 20 and 60 min. Following each interview, verbatim transcripts were produced using Happy Scribe (36); the second author performed quality checks of each transcript to ensure accuracy. Names and other identifying information were removed from transcripts.

### Data analysis

Data analysis consisted of a thematic analysis using MAXQDA Analytics Pro 2022. Thematic analysis refers to, “the method for recognizing, analyzing, and reporting patterns (themes) within data” (37). These phases involved data familiarization, initial code creation, theme search, theme review, and theme definition and naming (37).

An initial codebook of 16 deductive codes was developed using domains from the IDI guides and IPV literature. Next, the second author read through the dataset multiple times to become familiar with the data and develop memos. Inductive codes were developed as part of the data familiarization and preliminary memoing processes. Inductive codes were further developed based off recurring topics from interviews. Examples of deductive codes included “individual barriers for help-seeking” and “institutional barriers to care coordination,” while inductive codes such as “financial ties to abuser” and “lack of flexible funding” were developed based on recurring topics in the interviews.

Transcripts were then coded by a single member of the research team with team discussions about code application, inductive code development and theme development occurring weekly. During the coding process, the research question was kept in mind, focusing on barriers in the transition from hospital to community-based care among IPV survivors. The research team kept detailed memos throughout the coding process to document analytic decisions, potential themes, and reflections on the data. The initial 16 deductive codes were applied to all transcripts; 38 inductive codes were later added and organized in a hierarchical coding scheme and applied as needed to each transcript. This method aligns with Bazeley’s (38) approach to organizing code structures based on conceptual similarities, while also ensuring that each concept only appeared in the code structure once. The finalized codebook was then used to recode the first transcript and subsequent 13 transcripts.

Themes were developed based on the frequency and salience of codes across the dataset. Code co-occurrences and relationships between codes were explored to identify overarching patterns and themes. Themes were iteratively reviewed and refined to ensure they captured the most meaningful and coherent patterns in the data, while also considering their relevance to the research question and potential implications for practice and future research in IPV care coordination. The final themes were selected based on their prevalence across the dataset, the depth and richness of the data supporting them, and their ability to provide new insights into the barriers and facilitators of care coordination for IPV survivors transitioning from hospital to community-based services. Descriptive statistics were calculated using Qualtrics and Google Sheets to characterize the sample and provide context for the qualitative findings.

### Reflexivity statement

The research team consisted of individuals with expertise in public health, and qualitative research. Three identify as cisgender heterosexual women while the other is a cisgender heterosexual man; two team members identified as having a disability and one team member had lived experience of IPV. Throughout the research process, the team engaged in ongoing reflexivity to consider how their own experiences, assumptions, and biases might influence the data collection, analysis, and interpretation. Regular team meetings provided opportunities for open discussion and critical reflection on the emerging findings and the researchers’ positionality. The team also sought feedback from colleagues and stakeholders to challenge their assumptions and ensure the credibility and trustworthiness of the findings.

## Results

Participants (*N* = 14) included 13 women and one man who were staff of CBOs serving IPV survivors in Metropolitan Atlanta (Table 1). Of the 14 participants, 50% (*n* = 7) were Black or African American, 29% (*n* = 4) were White, and 21% (*n* = 3) identified their race as Other. The mean age of participants was 48 years. All participants completed higher education, with 14% (*n* = 2) completing a professional degree (MD, JD, etc.), 35% (*n* = 5) a bachelor’s degree, 42% (*n* = 6) a master’s, and 7% (*n* = 1) a doctoral degree. Participants saw an average of 16 IPV survivors per day. Participants worked at CBOs in six of the eleven counties that make up Metropolitan Atlanta: Cherokee, Clayton, Cobb, DeKalb, Fulton, and Gwinnett. Participants’ professional titles included: executive director, program director, manager, program coordinator, legal advocate; one police officer was also included. Participants had an average of 14.5 years of experience ranging from less than 1 year (0) to 39 years. All but one participant worked directly with IPV survivors; the outlier previously worked directly with survivors and at the time of the interview served in a leadership role at a CBO.

**Table 1:** Demographic characteristics of community based-organizational staff (*N* = 14).

Demographic	*N* = 14
*Age in years*	
Average	47.86
SD	9.5
*Self-reported gender, n (%)*	
Woman	13 (93%)
Man	1 (7%)
*Race, n (%)*	
Black or African American	7 (50%)
White	4 (28.6)
Other	3 (21.4)
*Highest level of education achieved, n (%)*	
Bachelor’s degree	5 (35.7%)
Master’s degree	6 (42.9%)
Doctoral degree	1 (7.1%)
Professional degree (JD, MD)	2 (14.3%)
*Years in social service, mean (standard deviation)*	
Average	14.5 (11)

Three inductive themes were developed using the data. Staff of community-based organizations serving IPV survivors perceived that: (1) IPV survivors face individual-, organizational-, and systems-level barriers during help seeking and service provision; (2) Care coordination between hospitals and CBOs is limited due to siloed care provision; and (3) Care coordination can be improved through increased bidirectional efforts.

### Theme 1: IPV survivors face individual-, organizational-, and systems-level barriers during help seeking and service provision

CBO staff identified a wide range of barriers that prevent IPV survivors from receiving needed services. These included individual-, organizational- and systems-level factors.

#### Subtheme 1.1: Individual-level barriers for IPV survivors

Individual factors noted by participants included: emotional ties and financial dependence on abusers as well as a lack of awareness about what constitutes abuse. Participants noted how the complex emotional bonds between survivors and their abusers can make it difficult to seek supportive resources or leave a relationship. These emotional ties are often intertwined with economic dependence. Participants observed survivors’ fears about abusers following through on threats resulting in vacillation between survivors’ desires for safety and “changes of heart” including recantation and choosing to stay in the relationship. Participants also noted that survivors may also have limited awareness of awareness of available resources—sometimes because of abuser’s isolation or coercive control tactics. Participants believed that many survivors, especially those experiencing IPV for the first time, may not recognize the full scope of abusive behaviors resulting in the normalization of abuse. One participant shared:

Many times my clients will say, ‘I thought that was normal’ or they will minimize what they’ve been going through and not realize that is a truly abuse. ‘Oh no, it’s nothing. It was just a small bruise, he just hit me once’.

Trauma and the psychological toll of abuse were also noted as factors impacting survivors’ ability to make decisions, assess relationship risk, or follow through on any plans to leave. The shame of IPV experience, stigma and potential loss of autonomy associated with IPV disclosure were also noted by participants as important individual factors.

Finally, and most relevant to care coordination, participants noted how individual circumstance may affect the ability of survivors to navigate complex health and social support systems. One participant shared:

It’s exhausting to a survivor. And I don’t feel like just doling out resources or giving [her], ‘here’s a bunch of places to go or call.’ She’s got her kids, she’s got to navigate… There’s so much going on and I think we must sometimes forget what it must feel like to be in her shoes. And so, I think we need better wraparound services.

The labyrinth of legal, medical, housing, and social services that IPV survivors must navigate to get help can be overwhelming and frustrating, leading some to give up. The sheer volume of steps and hurdles can feel insurmountable for survivors already grappling with trauma and limited resources. One participant captured survivors’ frustration:

And sometimes you have to go through two or three numbers to get to where you need to be. And people get frustrated and give up sometimes.

#### Subtheme 1.2: Organizational-level barriers for IPV service provision at community-based organizations

Participants identified competitive siloing and resource limitations as major organizational barriers to IPV service provision. Persistent barriers to effective collaboration and coordination included siloed approaches and competition rather than cohesive systems. One participant noted how such competition impedes meeting survivor needs:

I see a little bit of competition sometimes where that’s the feeling that we get, where I don’t feel like the victim’s needs are really the ultimate priority… And I just feel that agencies should really work better and have better trust between each one another and with the singular goal of just meeting that client’s needs in their time of need.

Some participants recommended exploring comprehensive, co-located service models such as Family Justice Centers that provide wraparound services through a centralized process. While recognizing challenges related to confidentiality and logistics, participants felt improved service integration could improve access and reduce burdens on survivors. One participant described:

Basically, there’s this concept where you take every stakeholder that would assist the victim of domestic violence and you put them all in one place. And that makes a lot of sense because when you have too much space between us, things get lost. And we don’t get to improve our processes if we never review our processes.

Finally, insufficient and inflexible funding emerged as a common barrier to providing comprehensive services. Participants noted that funding is often restricted and cannot be used for critical expenses such as transportation, childcare, and housing deposits that could significantly aid survivor independence. One advocate stated:

I think funding is our number one barrier and I would qualify that with saying it’s flexible funding because we do have donor funding that is earmarked for specific purposes and it’s very, very strict and we cannot use those funds for something that we may consider priority for our clients. We really don’t have enough of flexible funding.

#### Subtheme 3.3: Systems-level barriers to IPV service provision

Two major system-level barriers were noted by participants as negatively affecting IPV service provision: (1) the lack of safe and affordable housing; and (2) health care access and affordability.

First, the lack of safe and affordable housing options for IPV survivors came up universally as a major gap and source of frustration. Both temporary emergency shelter and permanent housing were mentioned. One participant described:

Right now, the biggest barrier is seeking shelter or finding shelters that have space available. That’s the biggest barrier right now. A second barrier is that most of the counties are not accepting new applications for housing vouchers, emergency housing vouchers.

Second, participants identified lack of health insurance coverage and concerns about medical costs as significant barriers that prevent many IPV survivors from seeking or receiving care. A participant explained:

A lot of our clients do not have access to Medicaid or any kind of care of that nature. And so, to be able to have the financial resources to be able to seek some of the care, it can be kind of an impediment.

While some CBOs attempt to assist survivors with medical bills, participants indicated that larger systemic changes are needed to ensure survivors can access essential healthcare without incurring crushing debt or compromising their safety.

### Theme 2: Care coordination between hospitals and community-based organizations is limited due to siloed care provision

#### Subtheme 2.1: CBOs receive few hospital referrals and these survivors have distinct needs

The majority of participants noted that very few of their clients come directly from hospital settings to their organizations. Overall, the percentage of survivors referred directly from hospitals to CBOs was reported to be low. A client specialist shared, *“Personally, since I’ve been working, since February of this year, I have not had any client that has come directly from an emergency room.”* One organization with an informal hospital partnership reported that between 15 and 20% of their clients come via hospital referral; when asked to estimate the proportion of clients that come from hospitals, all other participants reported percentages lower than this figure. One participant shared:

Well, I think sometimes people are in the moment, they’re in a crisis in the moment. And I would say that there’s a percentage of our clients that have to go to the hospital in the moment, but once their initial needs are met, then depending on what their situation is, they maybe will return to the abuser… I would say I’ve had clients definitely that went to the hospital, and I was expecting to see them, somebody in their family might have advocated for them, and I was expecting to see them in my office the next day to try to do the next steps, to try and do a protective order or try and find transitional housing or whatever it is. And then the person sort of just drops off the radar….

When describing the needs of survivors referred following hospital-based care participants described their needs as distinct from other survivors including the need for follow-up medical care, therapeutic treatment (e.g., physical therapy), and supportive services (home health care). Participants noted that few CBO staff have medical training, and that their organizations are not designed to nor do they have the capacity to provide these types of care. However, many participants noted that injuries requiring hospital care act as an alert as to the urgent needs of survivors as described by one participant, *“they are harmed already, so they shoot to the top of our priority list as far as trying to place them in a shelter.”*

#### Subtheme 2.2: CBO and hospital staff cross training is needed

Participants noted that while CBO staff do not have medical training, hospital staff are not well informed about how to manage IPV cases nor do they know of available IPV services. One participant noted that hospital staff often seem unaware of available community resources, suggesting they should consult with CBOs to facilitate appropriate referrals and transitions for survivors upon discharge.

I don’t know that sometimes it seems like there’s a disconnect between what the hospitals know is available within the community. So, you would assume that the social workers or nursing staff or other staff members in the hospital would have like our agency has a resource guide and when people call, whatever resources they need, we try to facilitate.

The need for more robust training to help healthcare providers recognize and respond to IPV was a recurring theme. Insufficient training was seen as leading to missed opportunities for intervention and referral, ultimately affecting survivor outcomes. One participant described:

I guess if you had to think of an overarching barrier, that’s probably it, which is that that hasn’t been their purview for so long. And from medical perspective that the providers are thinking our job is to treat the acute injury they don’t necessarily have in their training, their traditional training, that soft skill of how do we deal with someone who needs support and services beyond that?

#### Subtheme 2.3: Siloing acts as a barrier to care coordination between hospitals and CBOs

Participants identified numerous barriers hindering smooth coordination and continuity of care for IPV during referral, transitions from hospital to CBO services, and follow up.

Participants described agencies working in silos, with insufficient sharing of survivor information and follow-up after referrals. One participant stated:

I sometimes feel like we all work in silos, so we might get a call from somebody… She’s going to the hospital, let them know about us, and then they reach out to us. And then if they leave the hospital and come to us, it’s like that communication now has stopped because, ‘Okay, she’s out of our care now. Now y’all have her’.

A program coordinator further explained:

If the referring agency do not have good information on the client and you are not able to client at the point of contact, then that might affect the case management for the client, but it could also affect the outcome of that case management.

One participant identified lack of direct communication and contacts as key barriers:

The main barriers between hospitals and community-based organizations? Probably correspondence… I think it’s hard to get in touch with the right people at the hospitals when you really need them.

Several participants highlighted the absence of “warm handoffs,” or direct, coordinated transfers of care from hospital to CBO providers, as a barrier to care coordination.

Honestly, I think it’s a warm hand off. So, like the idea of having an individual who doesn’t receive referrals and then who would follow up with the referring agency and then the person who would internally follow up with that client or that client … that’s probably like the biggest barrier is having somebody that’s consistent and it comes without being almost without being said.

Another participant noted, *“But it seems like a lot of times the hospitals do not do that extra step of trying to make sure that the person’s going from a safe environment to another safe environment or a medical environment or one that will be able to be supportive of their medical needs.”*

### Theme 3: Care coordination can be improved through increased bidirectional efforts

Participants offered a wide range of suggestions for ways to improve IPV prevention and response. These included: early prevention through school-based healthy relationship education; IPV stigma reduction via community awareness raising; police education on trauma-informed care; and increased support for *pro bono* legal aid. Participants also made recommendations specific to increasing or improving care coordination between hospitals and community-based organizations. These recommendations were largely centered on the use of survivor-centered approaches, improved interagency collaboration, and care coordination resource allocation such as through dedicated staff members whose purpose would be to coordinate care between hospitals and community agencies.

#### Subtheme 3.1: Care should be survivor-centered and use trauma-informed approaches to minimize re-traumatization during service delivery

Participants consistently emphasized the importance of centering survivor choices and autonomy. Participants described various strategies for reducing the risk of re-traumatization, such as allowing survivors to share their stories on their own terms, coordinating services to avoid repetition, and attending to basic needs before dealing with emotionally taxing matters. A transition coordinator described their approach:

I think the connection with the advocate can begin with ‘this is your space, this is your story, this is your voice.’

Participants stressed the critical importance of respecting survivors’ self-determination and not replicating abusive or harmful power dynamics during services delivery.

There’s just so many ways that gets stripped away from a victim… If you lead someone towards an option without understanding the context that they’re living in, you can make things worse rather than better.

Participants underscored the necessity for a full continuum of care, delivered in a culturally competent manner especially for survivors of color and immigrants.

#### Subtheme 3.2: The establishment of formal partnerships and protocols between hospitals and CBOs are necessary for increased collaboration and improved care coordination

Recognizing the need for more integrated, coordinated care, participants advocated for strengthening hospital-CBO partnerships through cross-training, warm handoff protocols, co-location of services, and improved communication channels.

One thing I’m always attempting to try to figure out is how we can get more integrated training, collaboration, and partnership between hospital entities and domestic violence organizations… So those are the things that are missing in terms of having that flow of information so that they can work together when there’s a victim that needs both services.

Another participant described their prior unsuccessful attempts at sharing resources:

Some of them, they already have like their own brochure with the national hotline or any other hotline that I’m not aware of. And then when I’m telling them this is the Georgia State hotline and if you connect with all the 46 certified shelters so that’s why it would be better for the victim to call us directly rather than calling the national on any other number that probably they are not going to be providing. But I think they have their own policies and they don’t include our information. Sometimes I want to share with them our posters and the material in English and Spanish and they’re like, ‘Yeah, no thank you, we already have ours’.

Several participants suggested establishing a “family violence center” that brings together all stakeholders who assist IPV survivors in one place, noting that having too much space between providers leads to things getting lost. Beyond strengthening partnerships with hospitals, participants also called for greater collaboration, resource-sharing, and streamlined processes among CBOs to better serve survivors. Some envisioned a centralized referral and case management system to facilitate warm handoffs and ensure survivors do not fall through the cracks when navigating multiple agencies. Others described the potential benefit of having dedicated IPV advocates staff in hospital settings to better connect survivors to community-based care.

## Discussion

Our findings highlight the multi-level barriers IPV survivors face in accessing community-based care following medical care, the limitations of existing hospital-CBO coordination, and opportunities for improvement from the perspectives of CBO staff. Participants identified individual, organizational, and systemic factors that impede IPV survivors’ ability to seek help and receive comprehensive services. These insights align with prior research on survivor barriers while uniquely capturing the challenges CBOs navigate in meeting their needs. CBO staff recognized that IPV survivors may minimize their abuse and only seek hospital care when absolutely necessary. This finding is consistent with the robust literature on IPV stigma and disclosure hesitancy (39–41). When survivors are admitted or choose to seek help at a hospital, IPV can be difficult to identify and, in many cases IPV is not disclosed. For survivors that did seek care in hospitals, inconsistent contacts and a lack of bi-directional communications between hospital and CBOs were identified as challenges in supporting survivors’ transitions. Hospital staff are often unfamiliar or poorly networked with community-based resources. Yet, EDs have the potential to play an important role in breaking the cycle of violence by facilitating connections to CBOs and ensuring that survivors’ medical and social support needs are fully met (42–45). ED-based interventions show promise in responding to IPV (46). Kendall conducted an intervention in an urban Level I trauma center (*n* = 360; mean age 32 years; 97% female, 74% non-white) where 96% of survivors felt increased safety up to 12 weeks after consultation with a CBO advocate and service referral (18). Ideally, once medical needs have been met, IPV survivors would connect with ongoing community social support services (47–49). However, participants mentioned how many IPV survivors were unaware of the available resources even when leaving a hospital; meanwhile, CBOs may passively rely on referrals from hospitals, without actively seeking to connect those released from hospital care into their programming (23, 24, 46). Among our participants most reported that few of the survivors they served came via hospital referrals. Several studies have found that inadequate organizational resources, staff burnout, lack of training, and poor integration with other community services interferes with quality services to IPV survivors (23, 50).

Participants suggested warm handoffs as a way to break silos and ensure IPV survivor connections to community-based care. Warm handoffs have been evaluated as a quality improvement tool for transitioning care albeit not within the field of IPV (51). Warm handoffs can be used to ensure secure and efficient referrals while also maintaining continuity of care thought they are most commonly used in the contexts of mental health and substance use disorder (52); there is scant literature on warm handoffs among IPV survivors (53). What does exist includes notable limitations. For example, Dichter’s primary data collection did not include the perspectives of stakeholders beyond survivors (e.g., there was no representation by hospital or CBO staff) and there was no identification of structural factors that would be essential to supporting care transitions (54) necessitating research in this area.

CBO staff expressed a desire to improve care coordination with hospitals to reduce the possibility of survivor retraumatization and to minimize the harmful effects of IPV. They also expressed the importance of keeping the survivor at the center of care. This aligns with Kulkarni’s findings on enhancing IPV services including providing empathy, supporting the empowerment of survivors, individualizing care, and maintaining ethical boundaries (23). Participants’ emphasis on the importance of trauma-informed, survivor-centered care is consistent with best practices for IPV services. However, their experiences reveal gaps in the implementation of these approaches across systems. Efforts to improve coordination must prioritize survivor autonomy, cultural responsiveness, and minimizing re-traumatization.

Participants also desired a “one-stop shop” where survivors could rest and care for their needs. A few participants mentioned a Family Justice Center (FJC) where survivors could get shelter, therapy, career counseling, conduct a job search, gain transportation to a safe place, get official documents, and help for children and pets. Duncan et al. highlighted the value of FJCs, noting that such centers bring a “multitude of organizations under one roof and eliminates the hurdles so many survivors must jump through” (55). The first FJC began in San Diego and saw a 95% reduction in domestic violence homicides after 15 years (56). The US Congress later recognized the importance of Family Justice Centers in Title 1 of the Violence Against Women Act (VAWA) in 2005 and allocated funding to create more FJCs, which are considered a part of best practice (56). FJCs have been found to increase CBO effectiveness, increase survivor safety and empowerment, reduce survivor fear, and reduce homicides (57–59). FJCs also address the challenges survivors face when travelling to multiple locations to file police reports, receive counseling, and to obtain other services (60). Efforts are underway to establish several FJCs in Georgia modeled after those in Tennessee which houses over a dozen FJCs. Three Georgia locales have begun the intensive planning process, including in the cities of Marietta, Macon, and Waycross, Georgia although there are no current efforts to develop an FJC serving Atlanta (61).

CBO staff reported difficulty in meeting IPV survivors’ material needs—including shelter, and financial support—even after connecting with CBOs. IPV survivors who leave abusive relationships often face housing instability and homelessness due to elevated housing costs, economic insecurity, damaged credit, and poor tenant history. In 2003, one study found IPV survivors were four times more likely to experience housing instability when compared to those who did not experience IPV (62). Similarly, a study of 110 survivors receiving CBO services in Georgia found that 38% percent reported homelessness after fleeing abuse, and 25% were forced to leave their homes due to financial problems or partner harassment (63). Such challenges have likely been exacerbated by increasing housing costs and inflation following the COVID-19 pandemic. Notably, participants highlighted survivors’ financial needs, both immediate and longer term. In 2005, a national telephone poll found that 64% of IPV survivors reported that their ability to work was affected by violence (64). Physical injuries contribute to absenteeism because of abusers’ intrusions at work, harassment, disruption to sleep schedules, and behaviors such as hiding car keys to make job retention challenging for survivors (65). Notably, healthcare costs for those experiencing abuse were 42% higher than for non-abused women (66). Such costs can perpetuate economic instability and dependency on abusers as was mentioned by our participants. The desire to support survivors’ financial needs was viewed by participants as in tension with CBO funding structures and mechanisms. This finding aligns with other research which found funding to be a top challenge in the provision of IPV services in North Carolina (24). Structural challenges for meeting IPV survivors’ material needs are thus a persistent problem across US settings.

Overall, participants believed that IPV survivor needs were often unmet, and they expressed the desire for additional community-based resources to support survivors short- and long-term needs. Mittal’s meta-analysis found that community-based interventions resulted in a decrease of IPV among survivors (67). Likewise, a randomized control study found that survivor-focused outreach can decrease the severity of PTSD, depression, and fear 1 year after the abuse compared to IPV survivors who did not receive the services (68). Moreover, survivors who also were connected with social supports were more likely to leave an abusive relationship underscoring the importance of connection to such services (68). Yet even with several studies noting the benefits of IPV survivor connection to CBO services, there are few documented programs linking IPV survivors from hospitals to CBOs nor have rigorous evaluations been published. Our findings contribute to the limited literature on warm handoffs and care coordination for IPV survivors by highlighting the perspectives of CBO staff and identifying specific barriers and opportunities for improvement in the context of hospital-to-CBO transitions.

### Limitations

There are several limitations to this study. For our purposes we considered all organizations serving IPV survivors in the community as CBOs. This included government agencies such as police. This study included one participant who was a police officer. This participant expressed opinion and perceptions which were sometimes substantively different from those of participants working on non-profit organizations. However, we reached thematic saturation. The police participant added richness to the breadth of comments reflected in the themes.

As with all qualitative research, results cannot be generalized to the entire population of IPV survivors. This study applies to Atlanta, Georgia though there may be transferable lessons relevant to other US locales. Findings from this study should be complemented by expanding data collection to incorporate more IPV CBO staff voices from across US.

## Conclusion

This study sought to explore, from the perspective of CBO staff, the perceptions of IPV coordinated care between hospitals and CBOs. Participants identified silos and inconsistent communication/relationships between hospital and CBOs as major barriers to care coordination. They also suggested that programs or interventions including warm handoffs may support care connection. However, warm handoffs for IPV have not been well documented or rigorously evaluated, and more research needs to be done in this area. Participants urged the importance of survivor autonomy and the need to reduce retraumatization by coordinating care. They suggested a Family Justice Center as a medium to center survivor needs and reduce administrative burden. Finally, participants identified the material needs of survivors—shelter and cash—as major barriers including the inability of their own organizations to directly provide such resources due to budget constraints.

The consequences of IPV are far-reaching and devastate survivors, their families, and communities. Although Metropolitan Atlanta has a robust networks of CBOs supporting survivors Georgia still ranks 31st nationally for women killed by men (69). In one study 40% of IPV victims killed by their abuser sought help in an ED 2 years before the fatal incident underscoring the importance that interventions based in EDs and hospital settings may have (70). As IPV continues to be a pervasive issue, this analysis suggests that formalizing partnerships between hospital and CBOs, including dedicating staff persons to coordinate care connections via a warm handoff program could improve survivor care connection; likewise, the development of a Family Justice Center would reduce survivor retraumatization. Improving care coordination will require a collaborative effort among policymakers, funders, healthcare institutions, and CBOs to prioritize survivor-centered approaches and invest in effective partnerships. Additional research is needed on such interventions designed to improve care coordination to ensure survivors needs are met.

## Data availability statement

The raw data supporting the conclusions of this article will be made available by the authors, without undue reservation.

## Ethics statement

The requirement of ethical approval was waived by Emory University Institutional Review Board for the studies involving humans because this study was deemed to be exempt due to its nature as public health practice. The studies were conducted in accordance with the local legislation and institutional requirements. Written informed consent for participation was not required from the participants or the participants’ legal guardians/next of kin because verbal informed consent was procured.

## Author contributions

DE: Conceptualization, Formal analysis, Investigation, Methodology, Resources, Supervision, Writing – original draft, Writing – review & editing. JP: Data curation, Formal analysis, Investigation, Writing – original draft, Writing – review & editing. KW: Investigation, Project administration, Writing – review & editing. LW: Methodology, Formal analysis, Writing – review & editing.
